# Response of Circulating Inflammatory Markers to Intermittent Hypoxia-Hyperoxia Training in Healthy Elderly People and Patients with Mild Cognitive Impairment

**DOI:** 10.3390/life12030432

**Published:** 2022-03-16

**Authors:** Zoya O. Serebrovska, Lei Xi, Lesya V. Tumanovska, Angela M. Shysh, Sergii V. Goncharov, Michael Khetsuriani, Taisia O. Kozak, Denis A. Pashevin, Victor E. Dosenko, Sergii V. Virko, Viktor A. Kholin, Oksana N. Grib, Natalie A. Utko, Egor Egorov, Anna O. Polischuk, Tetiana V. Serebrovska

**Affiliations:** 1 Department of General and Molecular Pathophysiology, Bogomoletz Institute of Physiology, 01024 Kyiv, Ukraine; ltumanovska@biph.kiev.ua (L.V.T.); angela@biph.kiev.ua (A.M.S.); goncharov@biph.kiev.ua (S.V.G.); khetsuriani@biph.kiev.ua (M.K.); kozak.taya@biph.kiev.ua (T.O.K.); den-win@ukr.net (D.A.P.); dosenko@biph.kiev.ua (V.E.D.); anyapol777@gmail.com (A.O.P.); sereb@biph.kiev.ua (T.V.S.); 2 Pauley Heart Center, Department of Internal Medicine, Virginia Commonwealth University, Richmond, VA 23298-0204, USA; 3 Lashkariov Institute of Semiconductor Physics, National Academy of Sciences, 41 Nauki Ave., 03028 Kyiv, Ukraine; virko@email.ua; 4 Department of Age Physiology and Pathology of Nervous System, Chebotarev Institute of Gerontology NAMS of Ukraine, 04114 Kyiv, Ukraine; victorkholin@yahoo.com (V.A.K.); ksuna.m.o@ukr.net (O.N.G.); natautko@yahoo.com (N.A.U.); 5 CELLGYM Technologies GmbH, 14193 Berlin, Germany; egorov@cellgym.de

**Keywords:** intermittent hypoxia, Alzheimer’s disease, neutrophil extracellular traps, inflammation, hypoxia inducible factor 1, cognitive Impairment

## Abstract

Intermittent hypoxia-hyperoxia training (IHHT) is a non-pharmacological therapeutic modality for management of some chronic- and age-related pathologies, such as Alzheimer’s disease (AD). Our previous studies demonstrated significant improvement of cognitive function after IHHT in the patients with mild cognitive impairment (MCI). The present study further investigated the effects of IHHT on pro-inflammatory factors in healthy elderly individuals and patients with early signs of AD. Twenty-nine subjects (13 healthy subjects without signs of cognitive impairment syndrome and 16 patients diagnosed with MCI; age 52 to 76 years) were divided into four groups: Healthy+Sham (*n* = 7), Healthy+IHHT (*n* = 6), MCI+Sham (*n* = 6), and MCI+IHHT (*n* = 10). IHHT was carried out 5 days per week for 3 weeks (total 15 sessions), and each daily session included 4 cycles of 5-min hypoxia (12% F_I_O_2_) and 3-min hyperoxia (33% F_I_O_2_). Decline in cognitive function indices was observed initially in both MCI+Sham and MCI+IHHT groups. The sham training did not alter any of the parameters, whereas IHHT resulted in improvement in latency of cognitive evoked potentials, along with elevation in APP110, GDF15 expression, and MMP9 activity in both healthy subjects and those with MCI. Increased MMP2 activity, HMGB1, and P-selectin expression and decreased NETs formation and Aβ expression were also observed in the MCI+IHHT group. There was a negative correlation between MoCA score and the plasma GDF15 expression (R = −0.5799, *p* < 0.05) before the initiation of IHHT. The enhanced expression of GDF15 was also associated with longer latency of the event-related potentials P330 and N200 (R = 0.6263, *p* < 0.05 and R = 0.5715, *p* < 0.05, respectively). In conclusion, IHHT upregulated circulating levels of some inflammatory markers, which may represent potential triggers for cellular adaptive reprogramming, leading to therapeutic effects against cognitive dysfunction and neuropathological changes during progression of AD. Further investigation is needed to clarify if there is a causative relationship between the improved cognitive function and the elevated inflammatory markers following IHHT.

## 1. Introduction

The lengthening of lifespan over the past century has led to a significant increase in morbidity of age-related diseases. Alzheimer’s disease (AD) is the most common form of dementia (~60–70% of cases) among the aging population, affecting 50 million people worldwide with declined cognitive ability [1]. The precise triggers of amyloid plaques and neuronal tangles formation and the subsequent neuronal death remain partially understood. AD may affect not only the brain, but also causes release of peripheral biomarkers, such as amyloid β (Aβ) and fractions of amyloid precursor protein (APP) in platelets [2], and an array of inflammatory factors [3], which may disclose potential pathophysiological mechanisms and provide an opportunity for early diagnosis of the disease. Chronic inflammation in the brain is one of the main mechanisms involved with AD during the neurodegeneration processes commonly observed in AD [4]. Oxidative stress, blood-brain barrier permeability, and mitochondrial stress in neurons are potential contributors to the harmful effects of the prolonged neuroinflammation during AD progression [5].

Among them, mitochondrial damage plays an important role in brain inflammation and potentiates the progression of AD. The damaged mitochondria may leak mitochondrial proteins and calcium into the cytoplasm and trigger apoptotic and necrotic cell death. The mitochondrial damage associated molecular patterns (mitDAMPs) are circulating biomarkers of mitochondrial damage [6], and when entering the blood, they become strong attractants for leukocytes. In addition, cytochrome C (CytC) is one of the formyl peptides of mitochondria and its level in blood plasma reflects the degree of inflammation and necrosis in the body tissues [7]. 

High mobility group box 1b (HMGB1b) is a potential target for treatment of inflammation in the central nervous system diseases [8]. Acting normally as a chromatin-binding protein, HMGB1b enters the matrix and blood and becomes a potent mediator of inflammation when the nuclear membrane is damaged. It acts alone or in combination with other pro-inflammatory molecules (e.g., DNA, LPS, IL-1α, IL-1β) [9]. Depending on the state of cysteine, HMGB1b interacts with different receptors in a fully reduced state, such as that in the nucleus, it forms a complex with CXCL12 and causes chemotaxis more intense than from CXCL12 itself. If two of the three cysteines form a disulfide bond, then HMGB1b1 acquires the ability to activate TLR4 and MD-2. Upon further oxidation, it loses the ability to activate receptors, mainly RAGE and TLR4 [10]. The content of HMGB1b in plasma can serve as a marker of the intensity of inflammation and the effectiveness of anti-inflammatory therapy [11].

Growth/differentiation factor-15 (GDF-15) is an anti-inflammatory factor and also named as macrophage inhibitor cytokine [12,13,14,15], and GDF15 expression increases in response to stress of various origins, especially those caused by mitochondrial dysfunction [16]. In addition to anti-inflammatory action, GDF-15 was recently shown to participate cell signaling through glial cell-derived neurotrophic factor (GDNF) and family receptor a-like (GFRAL) and cause appetite suppression that may lead to cachexia, which is one of the aggravating factors in late stages of cancer [17,18,19]. Although some suggested that GDF15 is not associated with AD, others demonstrated specific roles for GDF15 in the development of AD [20]. Elevated GDF15 was associated with a higher risk of AD and lower total brain and hippocampal volumes, greater white matter hyperintensity volume, and poorer cognitive performance [21,22]. Moreover, exogenous recombinant GDF-15 diminished antibody levels in microglial cell culture, and being injected in the brain parenchyma of 5XFAD mice also led to a decrease in Aβ plaques [23]. In addition, neutrophil extracellular traps (NETs) respond to high levels of pro-inflammatory cytokines and represent chromatin with inserted proteases released by an activated cell DNA network and in turn serve as a trap for bacteria and other antigen carriers. There are several references showing that NETs in brain tissue play an important pathogenic role in AD, producing microcirculation disorders, microthrombus formation, and association with the development of disease [24].

Recent studies have underscored an anti-inflammatory and/or antioxidant therapeutic approach to reducing the rate of development of the main AD symptoms [25,26]. Intermittent hypoxic training is a non-pharmacologic and systemic therapy that has a wide spectrum of beneficial effects against major diseases/disorders in humans, such as metabolic, cardiovascular, and neurodegenerative ailments. For example, intermittent hypoxic training resulted in augmentation of hypoxic sensitivity and significant decrease in blood concentration of DOPA in the patients with Parkinson’s disease [27], improved collective inspiratory muscle activity in patients with amyotrophic lateral sclerosis [28], increased cerebral blood flow in patients with heart failure [29], provided cardiovascular benefits in elderly people [30], decreased systolic blood pressure in patients with hypertension [31,32], upregulated erythropoietin [33,34], improved cognitive performance and quality of life in old people [35], alleviated surgery trauma [36], diminished oxidative stress [37], and normalized blood insulin levels in pre-diabetic patients [38]. Nevertheless, some pathological forms of intermittent hypoxia, e.g., severe obstructive sleep apnea (OSA), may induce destructive consequences in the body, including mitochondrial dysregulation, acidosis, altered mitochondrial membrane permeability, and impaired ATP biosynthesis [39], leading to impairment in attention, memory, and executive function and acceleration of AD development, along with cardiovascular injuries [40]. On the contrary, unlike OSA, the therapeutic applications of moderate and well-controlled sessions of intermittent hypoxia-normoxia or intermittent hypoxia-hyperoxia are capable of triggering an adaptive phenomenon, often called hypoxic conditioning or preconditioning, which could protect vital organs, including heart and brain against hypoxia or ischemia-induced lethal cellular damages [41,42,43,44]. 

In a recent publication, we demonstrated a significant improvement of cognitive functions in persons with mild cognitive impairment (MCI) after a 3-week course of intermittent hypoxic-hyperoxic training (IHHT), and the cognitive function improvement was accompanied with changes of few biomarkers of AD progression in peripheral blood [45]. Based on the previous study, we sought to gain additional mechanistic insights into the neuroprotective effects of IHHT by conducting the present study to further investigate a hypothesis that the positive effect of IHHT on cognitive function and circulatory AD markers may be mediated through downregulation of chronic inflammation. Accordingly, we examined the parameters of cognitive function, circulating inflammatory markers and AD biomarkers in healthy elderly people and patients with MCI 1 day and 1 month after the IHHT course.

## 2. Materials and Methods

### 2.1. Characteristics of Participants

Similar to research previously described [45], the present study received approval from the Ethics Committee of Chebotarev Institute of Gerontology, Kyiv, Ukraine (protocol #9, approval date: 13 May 2019). Twenty-seven subjects (ages 52 to 76 years) participated this study with signed informed consent and met the inclusion criteria described previously in details [45]. Among them, 13 healthy elderly subjects and 16 patients diagnosed with MCI were recruited from the Department of Aging Physiology and Pathology of Nervous System of the Chebotarev Institute of Gerontology. Since AD is known to disproportionately affect women [46,47], the majority of our small cohort also consisted of female patients with MCI. The healthy control subjects were selected to be age- and gender-matched to the MCI groups (Table 1). The diagnosis of MCI was according to the revised Petersen criteria [48], which included subjective cognitive complaint, objective evidence of cognitive impairment, absence of difficulties in functional activities of daily life, and no dementia. Objective cognitive decline was evaluated using the Montreal Cognitive Assessment (MoCA) test [49,50], with the cut off score of 25 to 19 [51]. The Clinical Dementia Rating scale used was 0.5 points [52]. The selected healthy patients and subjects with MCI were randomly assigned to either Healthy+Sham, Healthy+IHHT, MCI+Sham, or MCI+IHHT group. The normoxia sham versus hypoxia-hyperoxia training conditions actually used in each session was single-blind to the participants. Table 1 reports the anthropometric characteristics and baseline blood pressure of the subjects.

Data are presented as mean ± standard deviation (SD). IHHT, intermittent hypoxic-hyperoxic training; MCI, mild cognitive impairment; BMI, body mass index; SBP, systolic blood pressure; DBP, diastolic blood pressure.

### 2.2. Protocol of IHHT

**Figure 1:**
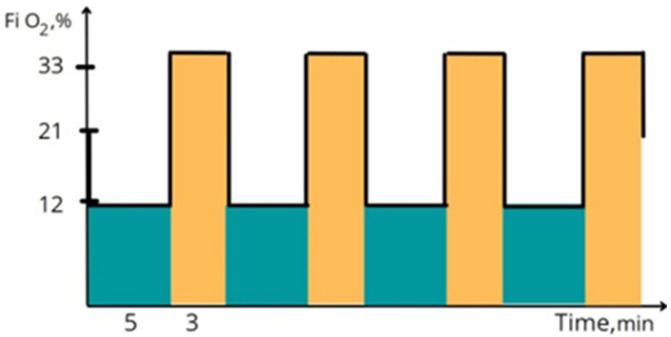
Illustrative description of the protocol for intermittent hypoxic-hyperoxic training (IHHT) sessions. Abbreviation: FiO_2_, Fraction of inspired oxygen levels.

All sessions of the present study were conducted in a quiet room at a comfortable temperature (21–23 °C). Venous blood samples were collected at three time points, i.e., (1) one day before the beginning of IHHT (for baseline values); (2) one day after completion of the 3-week sham or IHHT sessions (for immediate effects of IHHT); and (3) one month after the end of IHHT (for delayed effects of IHHT). Cell isolation of the collected blood samples was conducted immediately. Cognitive functions were investigated in all groups on the same day. The examinations were repeated one day and one month after the end of IHHT. The sessions of sham or IHHT were conducted five times a week for the subsequent 3 weeks, i.e., each subject received a total of 15 daily training sessions of sham or IHHT. As illustrated in Figure 1, each session of IHHT consisted of four cycles of 5-min hypoxia (12% inspired O_2_), followed by 3-min hyperoxia (breathing with 33% inspired O_2_) [53]. The IHHT sessions were performed using a computer-controlled apparatus of “CellAir One” (Cellgym Technologies GmbH, Berlin, Germany). During each session, we continuously monitored the subjects’ systolic and diastolic blood pressure, heart rate, and oxygen saturation.

### 2.3. Cognitive Function Assessment

MoCA test and long latency cognitive event-related potentials (ERPs) in the add ball paradigm were used for assessing brain function and cognitive status, as previously recommended [54,55,56]. ERPs were recorded in a soundproof room using 19-channel electroencephalography (EEG) equipment (Neurocom, XAI-MEDICA). The subjects were seated comfortably on an armchair and remained awake throughout the EEG test. The subjects were exposed to rare and frequent hearing stimuli delivered via speakers with 80 dB HL (decibels Hearing Level) sound intensity. ERPs were recorded at the maximum wave amplitude. Latency of N200 and P300 peaks of cognitive evoked potential in Cz electrode position were taken for analysis.

### 2.4. NETs Evaluation

NETs detection was performed as previously described [57]. The blood samples stabilized by EDTA were mixed with saline in 1:1 ratio and laminated on the Percoll gradient (45%, 54%, and 63% layer density). After 15 min of centrifugation (3000× *g* rpm), neutrophils were collected and purified, washed, and diluted in RPMI medium with 10% (*v*/*v*) heat-inactivated fetal bovine serum. Two sets of the neutrophils were placed in 24-well cell-culture vessels with a density of 100,000 cells/cm^2^ and incubated for 3 h. One set contained 20 nM of phorbol myristate acetate (PMA), which stimulates NETs formation (NETst). Another set did not contain PMA for studying spontaneously formed NETs (NETns).

### 2.5. Western Blot Analysis

The platelets were isolated as previously described [45] and then incubated in an ice-cold RIPA-buffer (1:3) containing 20 mM Tris–HCl, 150 mM NaCl, 1 mM EDTA, 1% NP-40, 1% sodium deoxycholate, 0.1% SDS, 1 µM leupeptin, and 1 mM protease inhibitor PMSF (pH 7.5) for 30 min and subsequently centrifuged for 15 min at 11,000× *g* at 4 °C, and the supernatant was collected. After measurement of protein concentration, the protein fraction of platelet lysate was separated using 10% polyacrylamide gel with 0.1% SDS and then transferred onto a nitrocellulose membrane (90 min at 200 mA). The membrane was incubated overnight at 4 °C with a purified mouse monoclonal primary antibody for APP (1:1000, MAB348, Sigma-Aldrich, St. Louis, MO, USA) or a rabbit monoclonal primary antibody for Aβ 1–42 (1:1000, ab201060, Abcam, Cambridge, MA, USA). In addition, a mouse monoclonal primary antibody for β-actin was used as an internal control for protein loading (1:2000, 2 h incubation, A1978, Sigma-Aldrich, St. Louis, MO, USA). Anti-rabbit or anti-mouse secondary antibodies were added to the membranes, which were visualized with chemiluminescence reagents (ECL kit, Amersham Pharmacia Biotech, Little Chalfont, UK) and exposed to X-ray film, and the scanned Western blot bands were analyzed with densitometry. Protein levels of APP and Aβ 1–42 were normalized against the corresponding β-actin values, and their relative expression ratio was calculated.

### 2.6. Enzyme-Linked Immunosorbent Assay (ELISA)

Plasma levels of HMGB1, CytC, TNF, P-sel, and GDF15 were determined using commercial kits as follows: HMGB-1 ELISA kit (Elabscience, Houston, TX, USA), CytC ELISA kit (Elabscience, Houston, TX, USA), Human TNFα High Sensitivity ELISA kit (Invitrogen, ThermoFisher Scientific, Wien, Austria), Human sP-selectin ELISA kit (Invitrogen, ThermoFisher Scientific, Wien, Austria), and Human GDF-15 ELISA kit (Thermo Scientific, Waltham, MA, USA), according to the manufacturer’s instructions. The absorbance signals of the ELISA were determined on a spectrophotometer (µQuant, Biotek, Winooski, VT, USA).

### 2.7. Analysis of MMPs Activity

MMP2 and MMP9 activity in plasma was assessed using a gelatin zymography in 10% polyacrylamide gel containing gelatin (2 mg/mL), as described [58]. Plasma samples were mixed in ratio 1:1 with non-reducing Laemmli’s buffer and subjected to sodium dodecyl sulfate-polyacrylamide gels co-polymerized with gelatin (2 mg/mL), followed by electrophoresis. Gels were washed twice with washing buffer and incubated overnight in developing buffer at 37 °C. Staining of gels was performed using 1% Coomassie Brilliant Blue G-250 (dissolved in an aqueous solution containing 10% acetic acid and 40% methanol) and unstaining with the same solution but without dye. MMP-2 and MMP-9 activity was detected as white bands on a dark background. Non-stained bands were shown on blue background gel revealing the gelatin digestion by MMPs at its respective molecular weight. The gels were scanned at 600 dpi and analyzed with densitometry using a Kodak Molecular Imaging PSB20.

The activities of MMP-2 and MMP-9 in plasma were evaluated using a gelatin zymography in 10% polyacrylamide gel containing gelatin (2 mg/mL). The samples were resuspended in non-reducing Laemmli buffer in ratio 1:1, loaded into a gel and underwent electrophoresis. After electrophoresis, the gel was washed with 50 mmol/L Tris-HCl (pH 7.4), containing 2.5% Triton X-100, and then incubated overnight at 37 °C in a developing buffer (50 mmol/L Tris-HCl, 10 mmol/L CaCl2, 1.25% Triton X-100, pH 7.4) at 37 °C. The gel was then stained with the solution, which contained 1% Coomassie Brilliant Blue G-25,040% methanol and 10% acetic acid, and de-stained with 40% methanol and 10% acetic acid solution. The gelatinolytic activities of the MMP-2 and MMP-9 were detected as white bands on a dark background. The intensity of these bands was assessed by a Kodak Molecular Imaging System.

### 2.8. Detection of Circulating lncRNA HIF1α-AS

Total RNA was extracted from the isolated platelets using the phenol–chloroform extraction method. RNA concentration was determined using a spectrophotometer (Model ND1000, NanoDrop Technologies, Wilmington, DE, USA). Reverse transcription of lncRNAHIF1α-AS1 was performed on 6 µg of total RNA using a RevertAidTM H Minus First Strand cDNA Synthesis Kit (Fermentas, Vilnius, Lithuania) with a random hexamer primer. The amplification of lncRNA HIF1α-AS1 was performed using SYBR Green PCR Master Mix with the following primers: F: 5′- GGTTGTTCATCTCGTCTCTGC -3′; R: 5′- CTTCTGGTTGGGGTACTGGAA-3′. The results were normalized with snRNA U6.

### 2.9. Statistical Analysis

The data were analyzed using IBM SPSS statistics software. Because the study design required three repeated measurements of the same subjects at the timepoints of the treatment, the data obtained were considered non-parametric. Accordingly, we applied Friedman’s test and Wilcoxon signed-rank test, which are non-parametric alternatives to one-factor repeated measures of the ANOVA, followed by a post-hoc Nemenyi test to assess the significance of difference among the groups. The Mann–Whitney U test was used to determine statistically significant differences between the groups. Correlation analysis was performed to determine the relationships between the measured parameters. Alpha value was set at 0.05.

## 3. Results

### 3.1. Cognitive Function

**Figure 2:**
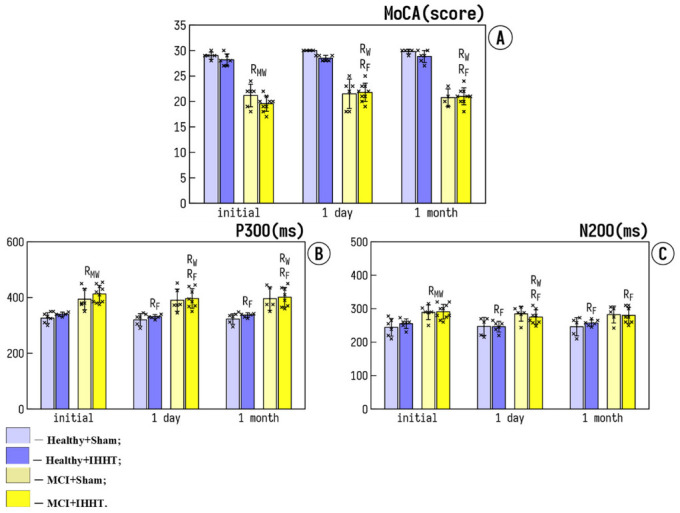
Effects of IHHT on cognitive parameters in healthy elderly controls and patients with mild cognitive impairment (MCI). The measurements were conducted at three time-points: Initial—the day before the initiation of sham or IHHT course; 1 day—one day after the termination of 3-week IHHT sessions; 1 month—one month after the termination of 3-week IHHT sessions. Bar 1—Healthy+Sham; Bar 2—Healthy+IHHT; Bar 3—MCI+Sham; and Bar 4—MCI+IHHT. (**A**) shows the Montreal Cognitive Assessment test (MoCA); (**B**,**C**) indicate the latency of N200 and P300 peaks of cognitive evoked potential. Data are presented as mean ± standard deviation (SD). Abbreviations: R_F_—significant difference (*p* < 0.05) compared to Initial by the Friedman test, R_W_—significant difference (*p* < 0.05) compared to Initial by the Wilcoxon test, R_MW_—significant difference (*p* < 0.05) between groups by the Mann–Whitney U test.

The baseline values of cognitive functional parameters were not different within either Healthy or MCI groups, but such a baseline differed significantly between Healthy and MCI groups. MoCA-test score was significantly declined in MCI+Sham and MCI+IHHT groups, as compared with Healthy+Sham and Healthy+IHHT groups (Figure 2A). The long latency cognitive evoked potentials (N200 and P300) were significantly higher in the patients with MCI (Figure 2B,C).

The sessions of sham treatment did not change cognitive parameters (i.e., Healthy+Sham and MCI+Sham groups), whereas a significant enhancement of the MoCA test score was observed in the MCI+IHHT group 1 day after the termination of IHHT. P300 and N200 latency were significantly reduced in Heathy+IHHT and MCI+IHHT groups, 1 day as well as 1 month after the completion of IHHT (Figure 2B,C). Thus, we observed improvement in cognitive function after the actual IHHT course both in people with MCI and in health participants, which means that IHHT improves the velocity of nerve impulses in healthy elderly individuals as well as patients with MCI.

### 3.2. Circulating AD Markers in Platelets: Amyloid Precursor Proteins (APP) and Amyloid Beta (Aβ)

**Figure 3:**
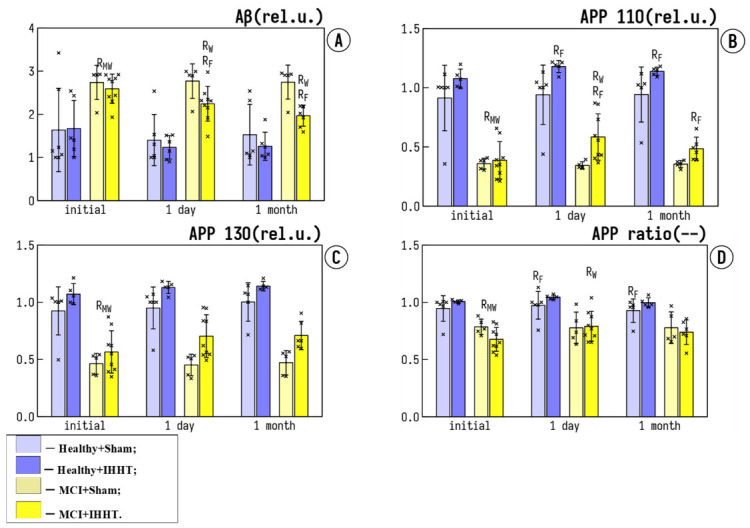
Effects of IHHT on Alzheimer disease-related markers in healthy elderly controls and patients with mild cognitive impairment (MCI). The measurements were conducted at three time-points: Initial—the day before the initiation of sham or IHHT course; 1 day—one day after the termination of 3-week IHHT sessions; 1 month—one month after the termination of 3-week IHHT sessions. Bar 1—Healthy+Sham; Bar 2—Healthy+IHHT; Bar 3—MCI+Sham; and Bar 4—MCI+IHHT. (**A**) shows expression levels of amyloid beta 1-42 (Aβ) in platelets; (**B**,**C**) indicate expression of APP130 and APP110 in platelets; and (**D**) shows the ratio of APP110/APP130 (amyloid precursor protein isoforms 110/130) in platelets. Data are presented as mean ± standard deviation (SD). Abbreviations: R_F_—significant difference (*p* < 0.05) compared to Initial by the Friedman test, R_W_—significant difference (*p* < 0.05) compared to Initial by the Wilcoxon test, R_MW_—significant difference (*p* < 0.05) between groups by the Mann–Whitney U test.

The basal Aβ expression in both MCI groups was significantly higher than in the healthy groups (Figure 3A). Sham training or IHHT did not significantly affect Aβ levels in Healthy+Sham, Healthy+IHHT, and MCI+Sham groups, whereas in the MCI+IHHT group, IHHT led to a significant decrease in Aβ levels 1 day and 1 month after the termination of IHHT. Similarly, the baseline levels of both 110 and 130 fractions of APP were significantly reduced in MCI groups as compared with the healthy controls (Figure 3B,C). The ratio of APP110/APP130 was shifted towards APP110 (Figure 3D). Neither APP130 nor APP110 changed in Healthy+Sham and MCI+Sham groups at 1 day and 1 month after the end of sham training sessions. On the contrary, IHHT caused an increase in APP 110, as well as the APP110/APP130 ratio in patients with MCI (MCI+IHHT group), and also elevated APP110 levels in the Healthy+IHHT group 1 day after the termination of IHHT, which returned to the baseline level after 1 month. We observed a significant improvement of circulating AD markers after IHHT sessions in both healthy elderly people and patients with MCI.

### 3.3. Circulating Inflammatory Markers

**Figure 4:**
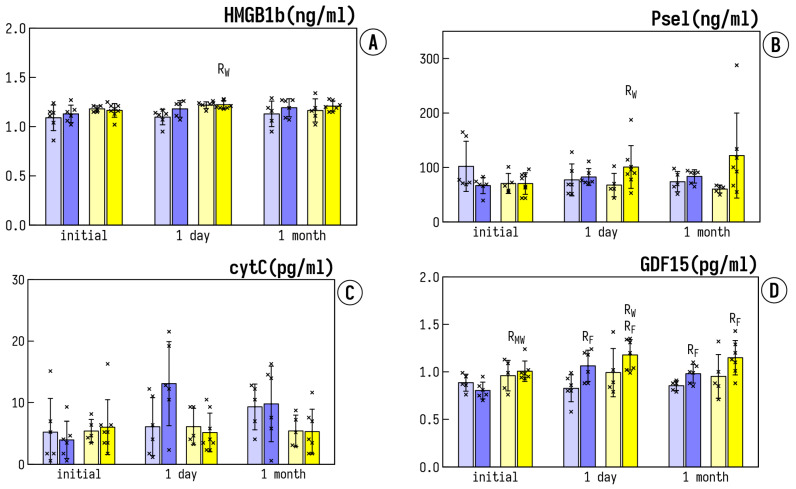

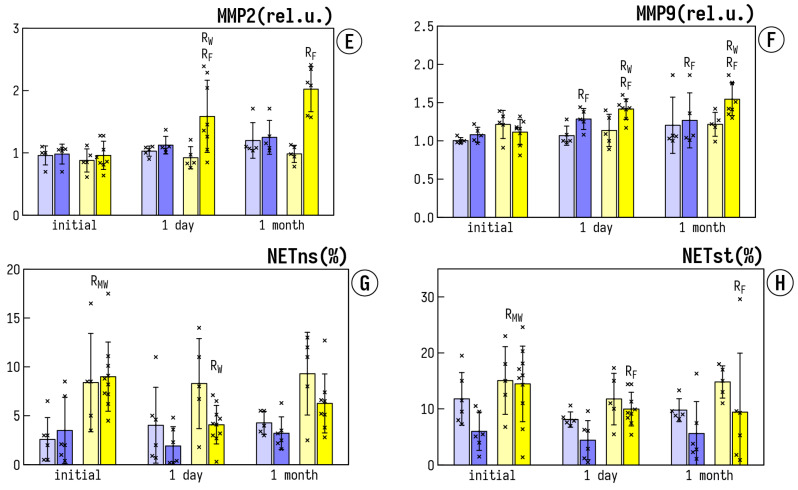

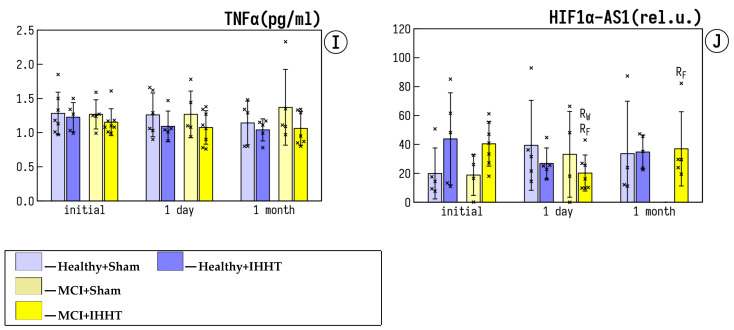
Effects of IHHT on circulating inflammatory markers in blood samples collected from healthy elderly controls and patients with mild cognitive impairment (MCI). The measurements were conducted at three time-points: Initial—the day before the initiation of sham or IHHT course; 1 day—one day after the termination of 3-week IHHT sessions; 1 month—one month after the termination of 3-week IHHT sessions. Bar 1—Healthy+Sham; Bar 2—Healthy+IHHT; Bar 3—MCI+Sham; and Bar 4—MCI+IHHT. (**A**) shows expression levels of High Mobility Group Box Protein 1 (HMGBP1b); (**B**) shows p-selectin (Psel); (**C**) indicates cytochrome C (cytC); (**D**) shows Growth Differentiating Factor 15 (GDF15); (**E**,**F**) indicate activity of matrix metalloproteinases 2 and 9 (MMP2 and MMP9); (**G**) shows neutrophil extracellular traps non stimulated (NETns); (**H**) shows neutrophil extracellular traps stimulated by phorbol miristate acetate (NETst); (**I**) shows expression of tumor necrosis factor α (TNFα); and (**J**) shows expression of hypoxia inducible factor 1α antisense long noncoding RNA (HIF1α-AS1). Data are presented as mean ± standard Deviation (SD). Please note that the data are missing due to a technical failure in RNA isolation for the assessment of HIF1α-AS1 at 1 month after the IHHT timepoint in the MCI+Sham group. Abbreviations: R_F_—significant difference compared to Initial by the Friedman test, R_W_—significant difference (*p* < 0.05) compared to Initial by the Wilcoxon test, R_MW_—significant difference (*p* < 0.05) between groups by the Mann-Whitney U test.

The baseline expression of HMGB1b was significantly higher in the MCI patients as compared to the healthy elderly people (Figure 4A). IHHT resulted in a further increase of HMGB1b 1 day after the termination of IHHT in the MCI+IHHT group. On the contrary, no changes occurred in both healthy groups or the MCI+Sham group at either 1 day or 1 month after the end of IHHT. P-selectin did not differ among any the groups at the baseline (Figure 4B). Sham or IHHT did not significantly affect P-selectin in Healthy+Sham, Healthy+IHHT, or MCI+Sham groups, whereas in the MCI+IHHT group, IHHT led to a significant increase in P-selectin 1 day after the end of IHHT (Figure 4B). There was no difference in CytC and TNFα expressions between all the groups, and no effect was observed after either sham or IHHT at any tested time-point (Figure 4C,I). The basal levels of GDF15 expression in both MCI groups were significantly higher than those of healthy controls (Figure 4D). There was a negative correlation between the MoCA score and the plasma GDF15 expression (R = −0.5799, *p* < 0.05) before the initiation of IHHT. The enhanced expression of GDF15 was also associated with longer latency of the event-related potentials P330 and N200 (R = 0.6263, *p* < 0.05 and R = 0.5715, *p* < 0.05, respectively). A less pronounced correlation between Aβ expression and GDF15 was observed, suggesting that the increase of anti-inflammatory factor expression seems to be a compensatory reaction to the chronic inflammation, which often accompanies MCI and AD development and influences Aβ accumulation. Whereas sham treatments did not alter significantly GDF15, IHHT led to a significant increase 1 day and 1 month after the termination of IHHT in both Healthy+IHHT and MCI+IHHT groups (Figure 4D). In addition, the basal activity of MMP2 and MMP9 was not different among the MCI groups or healthy groups and the sham treatment did not change these parameters in Healthy+Sham or MCI+Sham groups (Figure 4E,F). However, there was a significant enhancement of MMP2 and MMP9 induced by IHHT in the MCI+IHHT group and also MMP9 in the Healthy+IHHT group 1 day after the termination of IHHT (Figure 4E,F). The pronounced increase of MMP9 activity also sustained through 1 month after the finish of IHHT (Figure 4F). Finally, the baseline levels of non-stimulated NETs (NETns) were significantly higher in both MCI groups than those in Healthy groups (Figure 4G). Whereas sham training or IHHT did not significantly influence NETns in Healthy+Sham, Healthy+IHHT, and MCI+Sham groups, IHHT led to a significant decrease of NETns in the MCI+IHHT group 1 day and 1 month after the termination of IHHT (Figure 4G). A less pronounced but still significant decrease in the formation of PMA-stimulated NETs (NETst) was observed in the MCI+IHHT group 1 day after the end of IHHT (Figure 4H).

### 3.4. Expression of lncRNA HIF1a-AS1 in Plasma

Significant changes in expression of lncRNA hypoxia-inducible factor-1α-antisense RNA 1 (HIF1α-AS1), a blocker of HIF1α translation, were found (Figure 4J). Whereas sham training did not significantly affect HIF1α-AS in Healthy+Sham and MCI+Sham groups, IHHT led to a significant decrease in HIF1α-AS1 1 day after IHHT termination in both Healthy+IHHT and MCI+IHHT groups (Figure 4J).

## 4. Discussion

**Figure 5:**
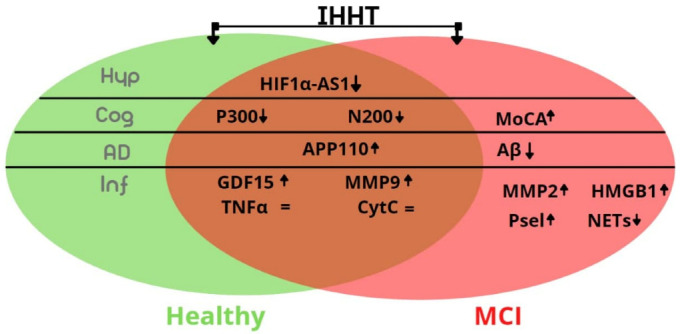
Summarized effects of IHHT on neurological and circulating parameters in healthy elderly participants (in green oval) and patients with MCI (in pink oval). Abbreviations: IHHT, intermittent hypoxia-hyperoxia training; MCI, mild cognitive impairment; Hyp, marker of hypoxia; Cog, cognitive related parameters; AD, Alzheimer disease related parameters; Inf, inflammation-related parameters; MoCA, Montreal Cognitive Assessment test; N200 and P300, latencies of N200 and P300 peaks of cognitive evoked potential; Aβ, amyloid beta 1-42 in platelets; APP110, amyloid precursor protein isoform 110 in platelets; HMGBP1b, High Mobility Group Box Protein 1b; P-sel, p-selectin; CytC, cytochrome C; GDF15, Growth Differentiating Factor 15; MMP2 and MMP9, matrix metalloproteinases 2 and 9; NETs, neutrophil extracellular traps; TNFα, tumor necrosis factor α; HIF1α-AS1, hypoxia inducible factor 1α antisense long noncoding RNA.

Intermittent hypoxia training/therapy has been increasingly investigated and used as a non-pharmacological and complementory therapeutic modality for a wide spectrum of human diseases and pathologic conditions, including cardiovascular, metabolic, geriatric, and neurodegenerative disorders, such as AD, whereas the cellular mechanisms underlying the beneficial effects remain partially understood. The salient finding of our present study is that, despite 3-week IHHT resulting in significant improvement in the latency of cognitive evoked potentials, not only in the patients with MCI, but also in the healthy participants, we did not observe a significant downregulation of circulating in-flammatory markers in the same participants studied, as summarized in Figure 5. 

Our next focus was on Aβ and APP, two major players in AD pathogenesis. The beneficial effect of IHHT was accompanied by a pronounced increase in APP110 fraction in platelets of both healthy participants and patients with MCI and a significant decrease in Aβ expression in the MCI patients one day after the termination of IHHT sessions. APP is concentrated in synapses, takes part in cell-matrix and cell–cell interaction in neurons [59], and participates in formation of synapses and synaptic plasticity [60]. This adhesion molecule is also involved in platelet hemostasis [61] and in sperm motility and sperm-oocyte interaction [62]. Abnormal APP expression in the patients’ platelets was similar to those found in their neurons [63,64]. Assuming APP expression in platelets serves as a valid peripheral biomarker of APP expression in the brain, the IHHT-induced improvement in cognitive function may be, at least partially, resulted from the increased APP content in the brain. 

Furthermore, the potential mechanisms of action of IHHT may include both direct cellular effects of oxygen deficiency and upregulation of HIFs and their downstream protein targets. lncRNA hypoxia-inducible factor-1α-antisense RNA 1 (HIF1α-AS1) is a long non-coding DNA that regulates HIF1α expression. It is located at the antisense strand of HIF1α of human chromosome 14, and the length of its mature body is 652 nt. [65,66]. In our present study, IHHT led to an increase of HIF1α-AS expression in patients with MCI, as well as healthy participants. Thus, it could be assumed that the observed effects of IHHT are at least partially related to the HIF1α cell signaling cascades summarized in Figure 5.

In addition, matrix metalloprotease 9 and 2 (MMP9 and MMP2) are collagenases that contribute to tissue remodeling by degrading extracellular matrix components, including the cleavage of cell surface ligands and chemokine/cytokine inactivation. The results of our current study showed that the activity of MMP9 and MMP2 in plasma significantly increases after IHHT training in healthy subjects and in patients with MCI. These findings were somewhat a surprise to us, since MMPs are pro-inflammatory elements and the elevation of their activities is associated with progression of pathological conditions, such as myocardial infarction. Elevated levels of MMP9 in brain were also found in patients with moderate and late stages of AD. Moreover, the Aβ-induced cognitive impairment was significantly reduced by MMP-9 gene silencing or treatment with MMP inhibitors [67]. MMP9 is able to bind and proteolyse LRP1 and LDLR and impair their ability to transport Aβ out of the brain [68]. Nevertheless, we postulate that the elevation of MMP2 and MMP9 activity may actually play an important mediator role in the cognitive function improvement by IHHT. This is because, firstly, MMPs are among the protein targets of HIF1α and are important players in angiogenesis under hypoxic conditions [69]. Additionally, MMP2 and MMP9 are produced in the brain by a variety of cell types, particularly by microglia and astrocytes, and they work together with insulin-degrading enzyme, neprilysin, and tissue/urokinase plasminogen activators to participate in Aβ hydrolysis at different cleavage sites [70,71]. 

It is noteworthy that our present study was designed for detecting the “chronic” or “stable” therapeutic effects of IHHT, and for this reason, we did not measure inflammatory markers immediately after each of the IHHT sessions. This difference in sampling timing may lead to the different results e previously reported by another group, who showed that exposure to 12% O_2_ for 30 min without re-oxygenation intervals caused activation of neutrophils characteristics, including phagocytosis [72]. Our present study also found a decrease in the NETs formation one day after IHHT in the MCI patients only (with increased baseline levels of NETs), but not in the healthy participants. Because NETs reflect the response of neutrophils to elevated inflammatory factors, the observed reduction in NETs may be resulted from a more intensive elimination of activated neutrophils from blood circulation. This finding was reinforced by the increased expression of P-selectin in plasma. P-selectin is expressed on the surface of stimulated endothelial cells, which binds with P-selectin glycoprotein ligand-1 expressed on the surface of circulating neutrophils, and in turn triggers neutrophil calcium flux and mediates its rolling and adhesion [73]. The migration of blood neutrophils and monocytes to the infected areas was highly dependent on P-selectin [74], and the spleen is responsible for rapid consumption of activated neutrophils [75]. Therefore, we suggest that IHHT could induce more rapid consumption of activated neutrophils in spleen via enhanced p-selectin expression. 

In our present study, patients with MCI were just at the beginning of their disease onset and no increases in TNFα and CytC levels were found compared to those of healthy control subjects, confirming the mild stage of AD in these patients. IHHT led to an unexpected increase in HMGB1, MMP2, and MMP 9 activity, as well as GDF15. Thus, IHHT may act as a “preconditioning” stimulus that causes the release of DAMPs into the extracellular environment, as shown in the increased levels of GDF15 in both IHHT-treated patients with MCI and healthy subjects. However, whether such a temporal association indicates a causative relationship or, alternatively, two parallel processes remains to be clarified in future investigations. Another apparent limitation of the present study is the very small sample size (*n* = 6 to 8 per group), mainly due to the difficulty in a single center setting to recruit more elderly patients with MCI who would comply for completion of the month-long IHHT protocol. Future multi-center studies with substantially larger numbers of participants would be ideal to further validate the key findings of the present investigation. 

## 5. Conclusions

The present study revealed that 3-week IHHT sessions resulted in significant improvement in the latency of cognitive evoked potentials not only in patients with MCI, but also in healthy elderly control subjects. The improvements in cognitive function were accompanied by significantly increased APP110 fraction in platelets and significantly reduced Aβ expression one day after the termination of IHHT sessions. Interestingly, IHHT also upregulated circulating levels of some inflammatory markers, which may represent the potential triggers for cellular adaptive reprogramming that led to therapeutic effects against cognitive dysfunction and neuropathological changes during progression of AD. However, whether these pro-inflammatory triggers play a causative role in mediating the beneficial effects of IHHT-induced neuroprotection remains to be determined.

## Figures and Tables

**Table 1:** Anthropometric characteristics of the participants.

Groups	N	Gender (Female/Male)	Age (Years)	BMI (kg/m^2^)	SBP (mmHg)	DBP (mmHg)
Healthy+Sham	7	5/2	65 ± 8.1	26.6 ± 3.8	133.8 ± 18.1	82.1 ± 8.0
Healthy+IHHT	6	5/1	67.5 ± 7	26.5 ± 4.7	129.0 ± 15.4	82.4 ± 8.0
MCI+Sham	6	6/0	70.8 ± 9.3	26.5 ± 4.3	136.4 ± 17.6	82.1 ± 14.0
MCI+IHHT	8	7/1	65.4 ± 6.2	26.8 ± 5.1	135.2 ± 15.4	82.7 ± 9.5

## Data Availability

All the data supporting the findings of this study have been provided.
